# The relationship between radiation dose and bevacizumab-related imaging abnormality in patients with brain tumors: A voxel-wise normal tissue complication probability (NTCP) analysis

**DOI:** 10.1371/journal.pone.0279812

**Published:** 2023-02-17

**Authors:** Mia Salans, Jordan Houri, Roshan Karunamuni, Austin Hopper, Rachel Delfanti, Tyler M. Seibert, Naeim Bahrami, Yasamin Sharifzadeh, Carrie McDonald, Anders Dale, Vitali Moiseenko, Nikdokht Farid, Jona A. Hattangadi-Gluth

**Affiliations:** 1 Department of Radiation Medicine and Applied Sciences, University of California San Diego, La Jolla, California, United States of America; 2 Carl E. Ravin Advanced Imaging Laboratories, Duke University, Durham, North Carolina, United States of America; 3 Department of Radiology, University of California San Diego, La Jolla, California, United States of America; 4 Department of Bioengineering, University of California San Diego, La Jolla, California, United States of America; 5 Department of Psychiatry, University of California San Diego, La Jolla, California, United States of America; Goethe University Hospital Frankfurt, GERMANY

## Abstract

**Purpose:**

Bevacizumab-related imaging abnormality (BRIA), appearing as areas of restricted diffusion on magnetic resonance imaging (MRI) and representing atypical coagulative necrosis pathologically, has been observed in patients with brain tumors receiving radiotherapy and bevacizumab. We investigated the role of cumulative radiation dose in BRIA development in a voxel-wise analysis.

**Methods:**

Patients (n = 18) with BRIA were identified. All had high-grade gliomas or brain metastases treated with radiotherapy and bevacizumab. Areas of BRIA were segmented semi-automatically on diffusion-weighted MRI with apparent diffusion coefficient (ADC) images. To avoid confounding by possible tumor, hypoperfusion was confirmed with perfusion imaging. ADC images and radiation dose maps were co-registered to a high-resolution T1-weighted MRI and registration accuracy was verified. Voxel-wise normal tissue complication probability analyses were performed using a logistic model analyzing the relationship between cumulative voxel equivalent total dose in 2 Gy fractions (EQD2) and BRIA development at each voxel. Confidence intervals for regression model predictions were estimated with bootstrapping.

**Results:**

Among 18 patients, 39 brain tumors were treated. Patients received a median of 4.5 cycles of bevacizumab and 1–4 radiation courses prior to BRIA appearance. Most (64%) treated tumors overlapped with areas of BRIA. The median proportion of each BRIA region of interest volume overlapping with tumor was 98%. We found a dose-dependent association between cumulative voxel EQD2 and the relative probability of BRIA (β_0_ = -5.1, β_1_ = 0.03 Gy^-1^, γ = 1.3).

**Conclusions:**

BRIA is likely a radiation dose-dependent phenomenon in patients with brain tumors receiving bevacizumab and radiotherapy. The combination of radiation effects and tumor microenvironmental factors in potentiating BRIA in this population should be further investigated.

## Introduction

The anti-vascular endothelial growth factor (VEGF) antibody bevacizumab is frequently added to standard therapy for high-grade gliomas, which are highly vascular and malignant [1, 2]. In brain metastases, bevacizumab is used to treat symptomatic radionecrosis [3]. In addition to targeting neoangiogenesis, bevacizumab normalizes blood-brain barrier (BBB) permeability, reducing vasogenic edema, decreasing steroid dependence, and alleviating clinical symptoms [4–6].

Tumors treated with bevacizumab often display a dramatic decrease in contrast enhancement soon after treatment initiation in a phenomenon known as pseudoresponse; yet, there is often residual viable tumor and even tumor progression. As a result, diffusion-weighted MRI (DWI) has emerged as an alternative method for monitoring recurrence [7, 8]. Specifically, apparent diffusion coefficient (ADC), a quantitative DWI metric that characterizes the random motion of water molecules within a voxel, inversely correlates with tumor cellularity and has been used as a marker of tumor progression [7, 9].

While DWI has improved detection of non-enhancing recurrent tumor, it has also led to the discovery of areas of restricted diffusion in patients receiving bevacizumab [10–12]. These areas, since described as bevacizumab-related imaging abnormality (BRIA) [13], are histopathologically distinct from traditional radiation necrosis, consisting of atypical coagulative necrosis rather than collagenous thickening [12] and fibrinoid necrosis [14]. Moreover, BRIA possesses a unique radiologic signature on diffusion and perfusion MRI. This was defined by our group in a previous study [13], and has aided clinicians in differentiating BRIA from residual or recurrent tumor in a noninvasive manner.

Multiple studies have examined BRIA’s onset, duration, and implications for survival; its pathophysiology remains less well understood [10–12]. Improved understanding of the factors contributing to BRIA development may further help to distinguish BRIA from tumor progression and radionecrosis in neuro-oncologic patients, who often present with unclear imaging findings. While BRIA is observed in patients receiving both bevacizumab and brain radiation, the cumulative radiation dose threshold for BRIA appearance is unknown. This is particularly relevant to the brain tumor population, who may receive multiple courses of radiotherapy. In a prior study [12], our group observed areas of restricted diffusion and hypoperfusion consistent with BRIA in brain areas receiving ≥60 Gy, suggesting that BRIA may occur in regions exposed to higher radiation doses. However, this study analyzed prescription dose rather than cumulative equivalent dose in 2 Gy fractions (EQD2) without coregistration of planning CT and ADC MRI images. Moreover, it did not include a voxel-wise normal tissue complication probability (NTCP) analysis of BRIA appearance similar to those used to explore radiation-mediated toxicities such as esophagitis and lung injury [15, 16]. In the present study, we examined the association between cumulative radiation dose and the voxel-specific likelihood of BRIA development. We hypothesized that higher cumulative radiation dose would be associated with a greater likelihood of voxel-specific BRIA development.

## Materials and methods

### Study characteristics and subjects

This retrospective study was approved by the Institutional Review Board at UC San Diego. All data were anonymized for analyses and the institutional review board waived the requirement for informed consent. Between March 2011-September 2019, we identified 29 patients with high-grade glioma or brain metastases who developed BRIA from a prospective list of patients receiving bevacizumab and brain radiotherapy maintained by our neuroradiology team. BRIA lesions were visualized on a post-radiation/bevacizumab magnetic resonance image (MRI) obtained using our standardized brain tumor imaging protocol. The presence of BRIA was confirmed by a neuroradiologist (N.F.) who initially defined the radiologic criteria for BRIA diagnosis [13]. BRIA was identified according to methods our group used to classify BRIA in our previous work [12, 13]; namely, the presence of restricted diffusion AND hypoperfusion in the same region of interest (ROI). Hypoperfusion was defined as mean relative cerebral blood volume (rCBV) <1.0. Patients with these imaging findings persisting on >1 MRI were keyworded in our institutional picture archiving and communication system. Patients with any concern for tumor progression at first BRIA occurrence (using multiparametric MRI sequences including perfusion imaging) were excluded to avoid potential confounding by tumor. As we sought to perform voxel-wise analyses, 11 patients were excluded due to unavailability of DICOM dose files for all radiotherapy courses prior to the first appearance of BRIA. Patient characteristics were ascertained from the medical record.

### MR imaging

MRI was performed on a 3.0 T GE Signa Excite HDx scanner equipped with an 8-channel head coil (GE Healthcare, WI, USA). The standardized brain tumor imaging protocol included pre- and post-gadolinium 3D volumetric T1-weighted inversion recovery–spoiled gradient recalled echo (IR-SPGR) sequences (echo time [TE]/repetition time [TR] = 2.8/6.5 ms; inversion time [TI] = 450 ms; flip angle [FA] = 8 degrees; field of view [FOV] = 24 cm; 0.93×0.93×1.2 mm), 3D T2-weighted FLAIR sequence (TE/TR = 126/6000 ms; TI = 1863 ms; FOV = 24 cm; 0.93×0.093×1.2 mm), and spin-echo echo-planar imaging (EPI) DWI sequence (TE/TR = 92-94/9500 ms; FOV = 28 cm; section thickness = 4 mm; matrix = 128×192). ADC volumes were calculated from acquired DWI data with *b* = 1000 s/mm^2^ and *b* = 0 s/mm^2^.

### ROIs

Treated tumor volume was defined as the planning target volume (PTV) from radiation dose plan records. BRIA ROIs were obtained from the first post-radiotherapy MRI on which BRIA was identified and assessed at a single timepoint. Areas of restricted diffusion were segmented semi-automatically (Amira software package, Visage Imaging) on the DWI sequence, and ADC images confirmed true restricted diffusion as areas of ADC hypointensity. All ROIs were drawn by two board-certified neuroradiologists (R.D and N.F.). Hypoperfusion was confirmed in the ROI, defined as mean relative cerebral blood volume (rCBV) <1.0.

### Image processing and registration

Using MIM version 7.0.4, ADC volumes and their corresponding BRIA ROIs were registered to a high-resolution T1-weighted sequence. For each treatment course, radiation dose maps and structure sets from treatment planning CT scans, including the brain and PTVs, were registered to T1-space, bringing dose volumes, structure files, and BRIA ROIs into a common space. Finally, all volumes in T1-space were resampled to a cubic voxel resolution of 0.5 mm in each direction.

Registration accuracy between CT and ADC MRI was evaluated using the mean distance to agreement (MDA) metric for the contours of well-defined brain structures. For each case, a physician contoured a normal structure (third or fourth ventricle, quadrigeminal cistern) on the ADC and each of the treatment planning CT images, blinded to the registration. For each CT, the MDA was calculated with respect to the ADC image. Of the values calculated for each subject, the maximum MDA was selected to represent a conservative estimate of registration accuracy.

### EQD2 analysis

Only dose records up to the first appearance of BRIA were included. To account for multiple radiation courses and fractionation schedules, the EQD2 within each voxel was estimated with Eq 1 and used to generate a single three-dimensional EQD2 volume for each patient,

$$EQD2=\sum_{i=1}^{Courses}\frac{D_{i}∙\left[1+\frac{D_{i}/n_{i}}{\alpha /\beta }\right]}{\left[1+\frac{2\;Gy}{\alpha /\beta }\right]}$$ (1)

where *D*_*i*_ is the dose at each voxel location per radiation course, *n*_*i*_ is the number of fractions per course, and α/β = 2 Gy [17].

### NTCP modeling

A logistic model described the voxel-wise incidence of BRIA as a function of cumulative EQD2. The data used to fit the model included voxels that fell within brain volumes for each patient. The relationship between the probability of BRIA within a specific voxel, X%, and cumulative EQD2 expressed as a logit transformation is:

$${EQD2}_{X}=\left[-\text{ln}\left(\frac{1}{\left(\frac{X}{100}\right)}-1\right)-\beta_{0}\right]/\beta_{1}$$ (2)

$$\gamma =-\beta_{0}/4$$ (3)

where EQD2_x_ is the EQD2 corresponding to an X% relative voxel-wise probability of BRIA, β_o_ is the log odds of BRIA at 0 Gy, and β_1_ is the change in log odds of BRIA per unit increase in dose. Normalized slope, γ, is the percent change in the probability of BRIA per 1% change in EQD2 at a 50% probability of BRIA.

The logistic regression analysis was performed using the Python [18] library scikit-learn [19]. To account for repeated measures, bootstrapping with 2000 samples estimated confidence intervals for model predictions. For each bootstrap sample, patients were randomly sampled with replacement to account for the small cohort, and voxels were sampled for each sampled patient with replacement to account for brain tissue’s stochastic response to radiation dose (i.e. if the same patient was sampled twice in a bootstrap sample, the voxel-level sampling ensured that the BRIA response to dose was not exactly identical). The upper and lower bounds of the 95% confidence interval for each EQD2 were determined by sorting the 2000 model outputs and taking the 50^th^ and 1950^th^ prediction.

### Progression and survival analysis

Per our institutional standard of care, patients with high-grade gliomas and with concern for tumor progression underwent surveillance MRI every eight and four weeks, respectively. Each patient was assessed for the presence and date of tumor progression on subsequent surveillance MRIs utilizing RANO criteria [20]. The location of progression was classified as either within or outside of the previously identified BRIA ROI. Kaplan-Meier analyses estimated median overall survival (OS) from the date of BRIA detection. The log-rank test compared survival between patients with primary versus metastatic tumors. Statistics were performed in R version 3.4.

## Results

### Patient cohort

**Fig 1:**
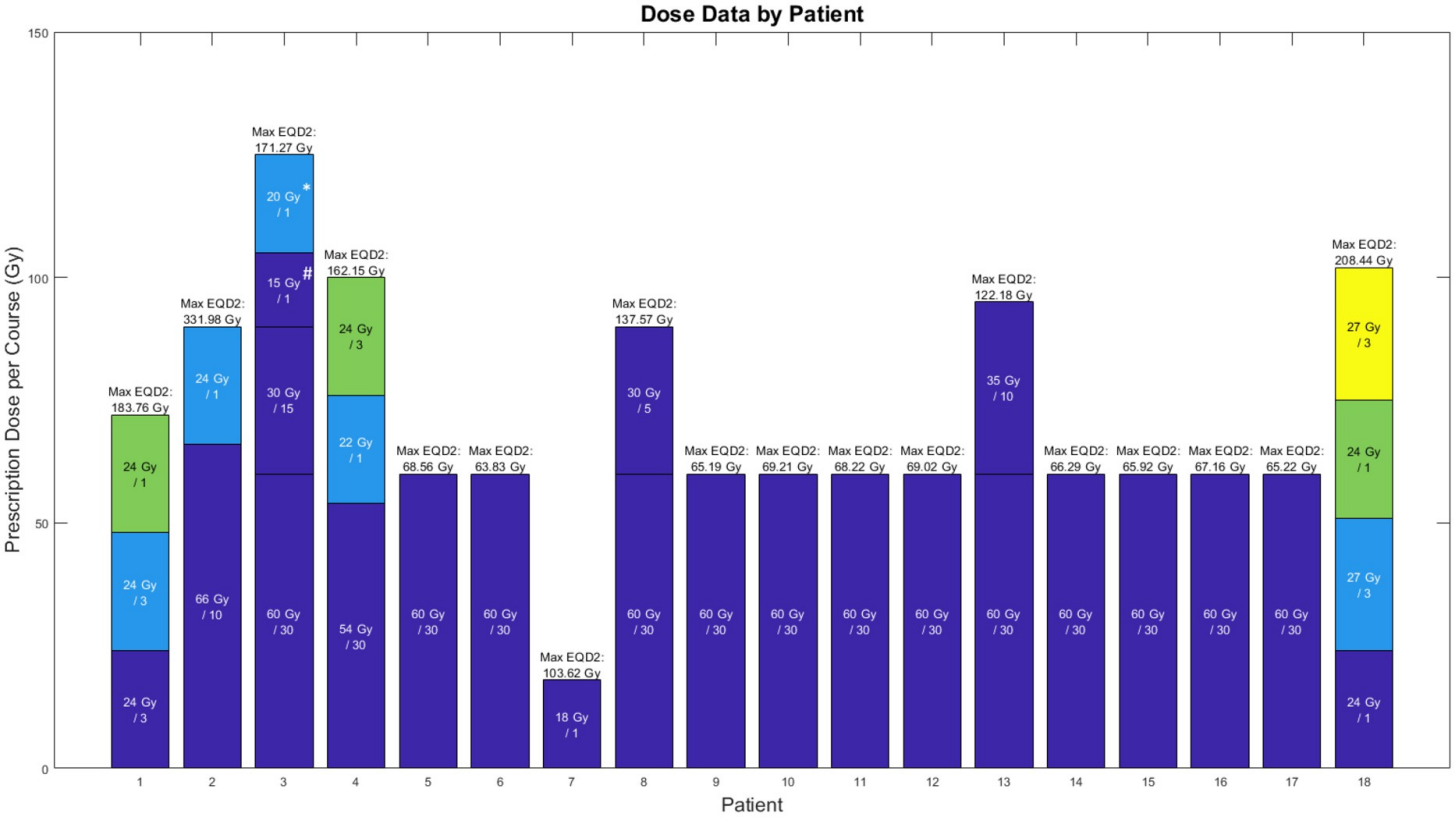
Radiation dose and course information. Radiation courses are represented by individual bars, where bar height indicates prescription radiation dose. Maximum voxel EQD2 for each patient is listed at the top of each bar. Distinct PTVs are color-coded. For example, patient 1 was irradiated at three distinct PTVs, while patient 3 received four courses of radiation to 2 distinct PTVs. #Patient 3, Course 3: this volume was in the Course 2 volume but not in the Course 1 volume. *Patient 3, Course 4: this course consisted of 3 separate PTVs: 1 overlapped with the original Course 1 volume; the other 2 were distinct. Abbreviations: EQD2, equivalent total dose in 2 Gy fractions.

Among 18 patients, 39 tumors were treated with radiotherapy. Most (69%) were high-grade gliomas. Patients received a median of 4.5 bevacizumab cycles prior to BRIA appearance. Patients received 1–4 courses of radiation with a median EQD2_max_ of 75.0 Gy, where EQD2_max_ represents the per-patient maximum EQD2 value. Dose data by patient is shown in Fig 1. Patient, tumor, and treatment characteristics are shown in Table 1.

**Table 1:** Patient, tumor, and treatment characteristics.

**Patients (n = 18)**	
Sex	
Male	10 (56)
Female	8 (44)
Median age at appearance of BRIA (y) (range)	58.5 (36–79)
Surgery	17 (94)
Biopsy	7 (50)
STR	4 (29)
GTR	3 (21)
Chemotherapy	17 (94)
Median number of radiation courses (range)	1 (1–4)
Median EQD2_max_ (Gy) (range)	75 (64.9–332.0)
Median cycles of bevacizumab prior to BRIA appearance (range)	4.5 (1–26)
**Treated target lesions (n = 39)**	
Median PTV volume (cc) (range)	10.1 (0.1–706.2)
Tumor type	
Original lesion	34 (87)
Recurrent	5 (13)
Tumor histology	
High grade glioma	27 (69)
Brain metastases	12 (31)
Tumor location (lobe)	
Temporal	4
Frontal	10
Parietal	4
Frontoparietal	7
Temporoparietal	2
Temporooccipital	1
Parietooccipital	1
Multilobar	7
Cerebellar	1
Corpus callosum	2

Abbreviations: STR, subtotal resection; GTR, gross total resection; EQD2, equivalent dose in 2 Gy fractions; EQD2_max_, per-patient maximum EQD2 value; PTV, planning target volume.

### Registration accuracy

Across all subjects, the median registration accuracy between ADC and CT was 0.23 mm (range: 0.052–0.896 mm). According to the AAPM TG-132 recommendations on image registration [21], the tolerance for acceptable accuracy of image registration using the MDA technique is to be within either the contouring uncertainty of the structure (2 mm for brain organs at risk) or the maximum voxel dimension (0.5 mm in this study). All subjects met MDA by contouring uncertainty, and all but 3 met MDA by voxel resolution < 0.5mm.

### BRIA characteristics

**Fig 2:**
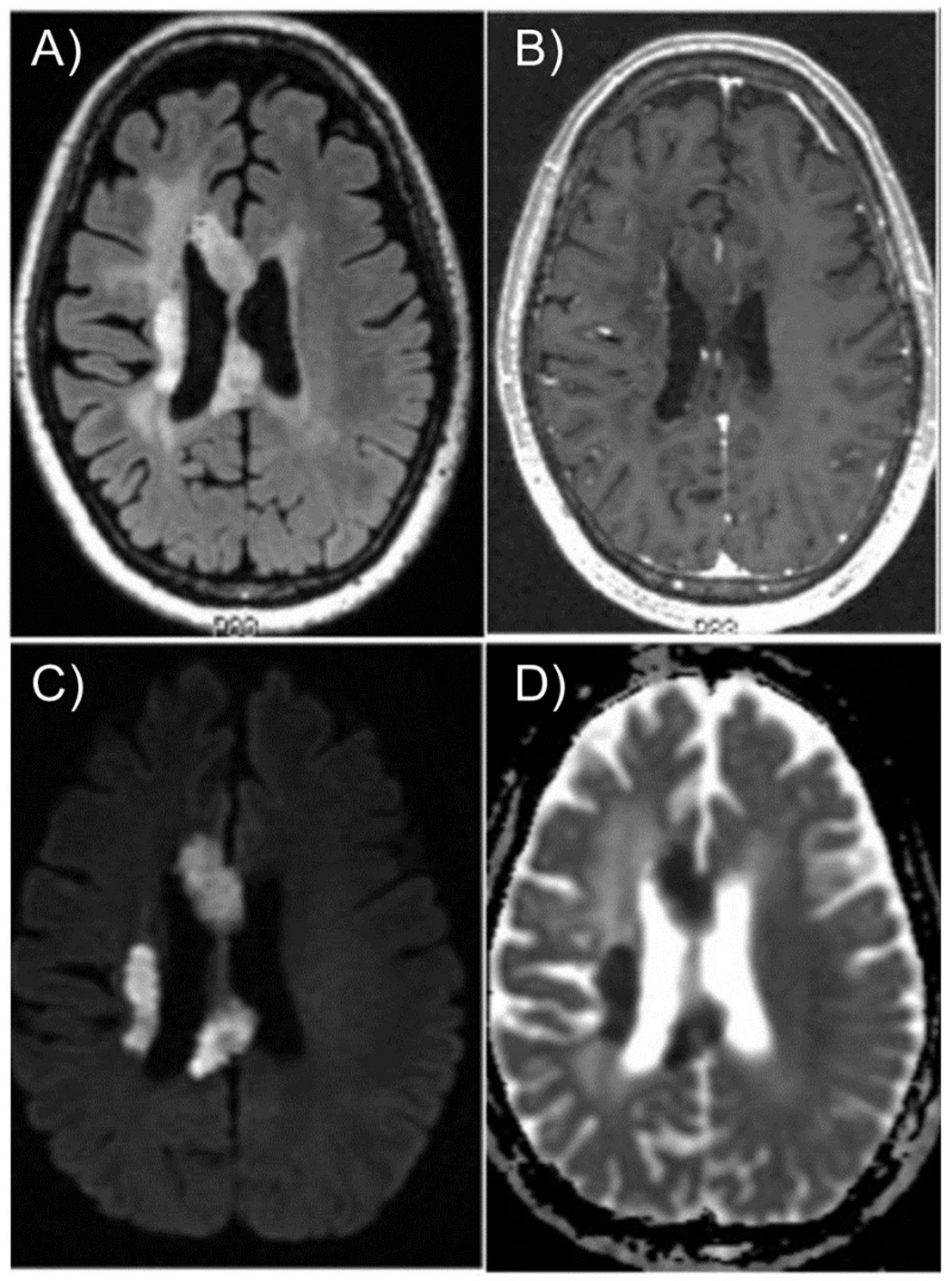
Example BRIA lesion. Appearance of BRIA in a 65-year-old male two months after starting bevacizumab for recurrent right parietal glioblastoma on a) FLAIR, b) T1 post-contrast, c) DWI, and d) ADC. Abbreviations: FLAIR, fluid-attenuated inversion recovery; DWI, diffusion-weighted imaging; ADC, apparent diffusion coefficient.

BRIA characteristics are shown in Table 2 with an example of BRIA in a patient case shown in Fig 2. Most (64%) tumors overlapped with BRIA when T1-weighted MRIs were overlaid with ADC images. The median proportion of BRIA ROI volume overlapping with tumor was 98% (range = 4.3%-100%). The median EQD2_max_ to overlapping and non-overlapping tumors was 100.8 (range = 63.8–332.0) and 98.3 (range = 63.7–162.2) Gy, respectively. The median time between the first radiation course to overlapping tumors and BRIA appearance was >1 year (S1 Fig). The median time between the first bevacizumab dose and BRIA appearance was 67 days (range = 2–645).

**Table 2:** BRIA characteristics.

**BRIA ROIs (n = 18)**	
Median ROI volume (cc) (range)	23.7 (8.7–99.0)
BRIA location (lobe)	
Temporal	3
Frontal	5
Parietal	6
Frontoparietal	2
Temporoparietal	1
Multilobar	1
**PTVs overlapping with BRIA (n = 25)**	
Histology of tumors corresponding to PTVs overlapping with BRIA*	
High grade glioma	22 (88)
Brain metastases	3 (12)
Median PTV-BRIA Dice metric for overlapping PTVs* (range)	0.1 (0.05–0.54)
Median percentage of BRIA ROI volume within any PTV (%) (range)	98.3 (4.3–100)
Median EQD2_max_ to overlapping PTVs* (Gy) (range)	100.8 (63.8–332.0)
Median EQD2_max_ to non-overlapping PTVs* (Gy) (range)	98.3 (63.8–162.2)

Abbreviations: ROI, region of interest; PTV, planning target volume; EQD2, equivalent total dose in 2 Gy fractions; EQD2_max_, per-patient maximum EQD2 value, RT, radiation therapy.

*Overlapping PTVs include PTVs that overlapped with BRIA ROIs when T1-weighted MRI images were overlaid with ADC images.

### NTCP modeling

**Fig 3:**
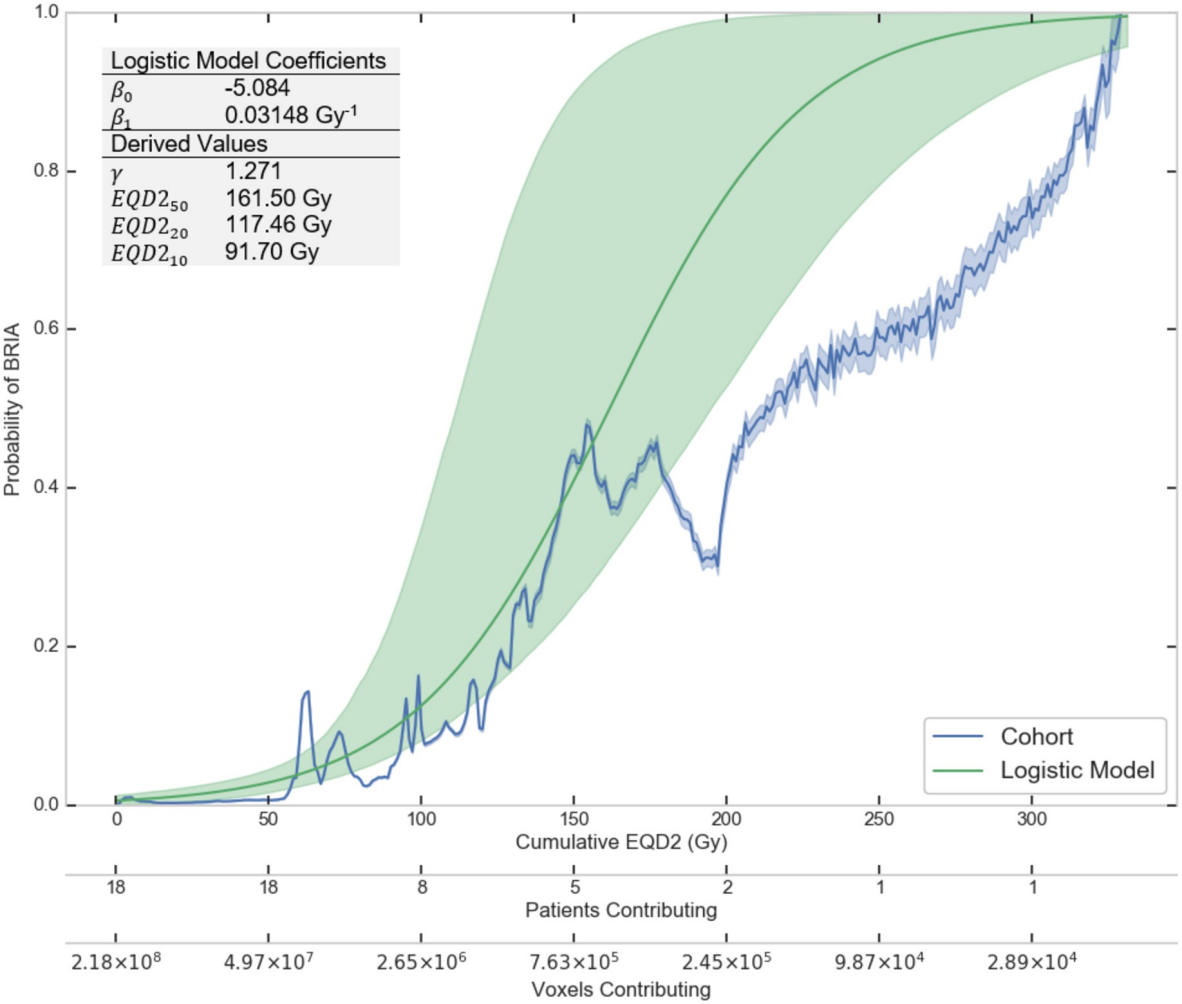
NTCP model of the relationship between radiation dose and probability of BRIA development. The relationship between cumulative voxel EQD2 (Gy) and the relative probability of BRIA as observed in this cohort (blue curve) and as calculated by the logistic regression NTCP model (green curve). The blue and green shaded regions represent the upper and lower bounds of the 95% confidence intervals for the observed probability of BRIA and the logistic model from bootstrapping, respectively. The figure insert shows EQD2 values for 10, 20, and 50% probability of BRIA derived from the model. Abbreviations: EQD2, equivalent total dose in 2 Gy fractions; γ, normalized slope of the dose-response curve; EQD2_x_, radiation dose corresponding to an x% relative probability of BRIA development.

**Fig 4:**
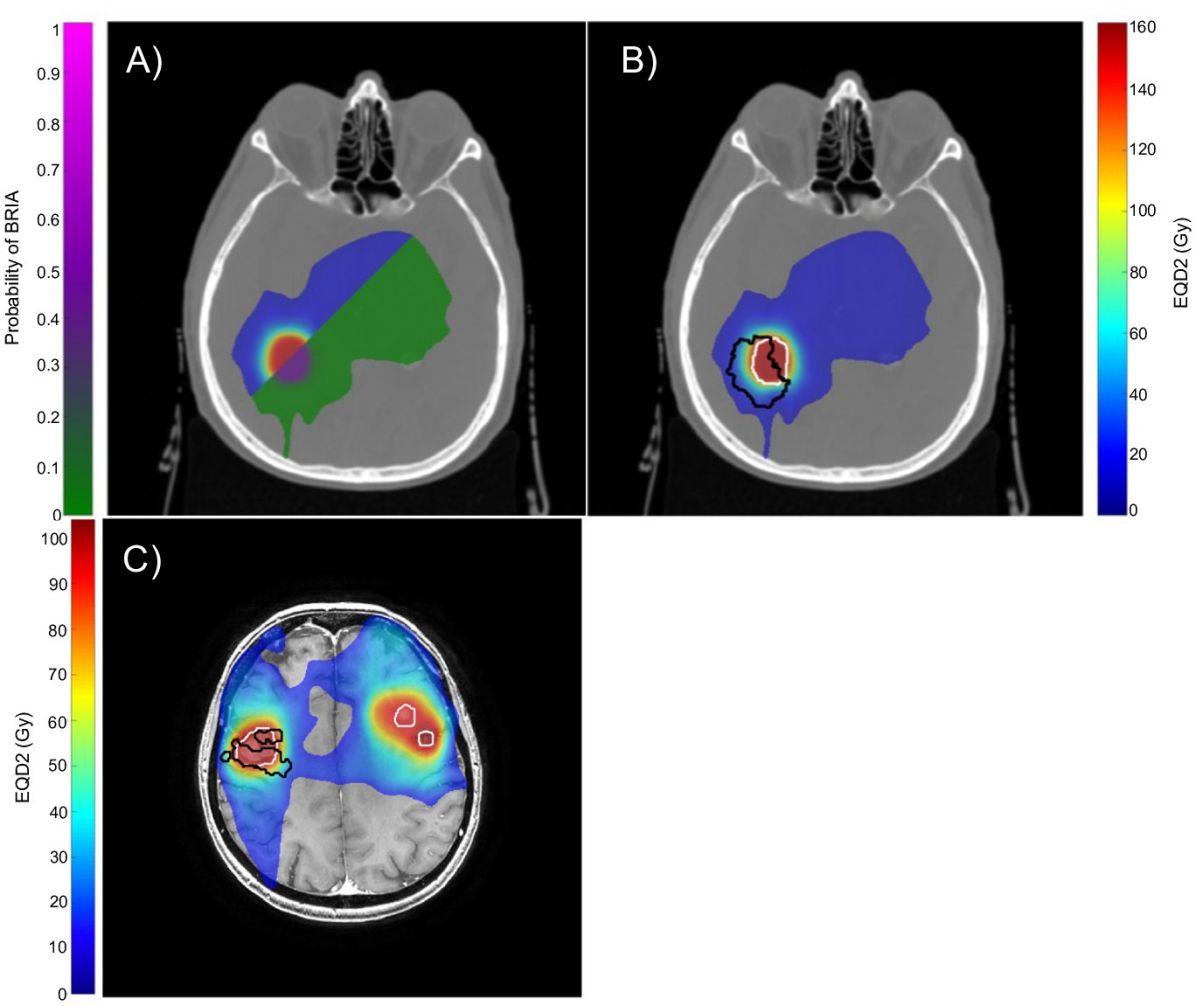
Agreement of the logistic model with the radiation dose map and observed BRIA ROI for two representative patients. Voxel EQD2 is represented by the blue to red scale. Panel A displays the cumulative voxel EQD2 map alongside the estimated probability of BRIA as predicted by the logistic regression model (green to pink scale) in a representative patient. Panel B demonstrates the cumulative voxel EQD2 map overlaid with the PTV (white) and the observed BRIA ROI (black) for the same patient. Panel C illustrates PTVs (white) and observed BRIA ROIs (black) in a second patient. The patient developed BRIA in one of three PTVs. Abbreviations: ROI, region of interest; EQD2, equivalent total dose in 2 Gy fractions; PTV, planning target volume.

The predicted relationship between cumulative EQD2 and the voxel-wise probability of BRIA was derived using estimates from the logistic model (Fig 3). The *β*_0_ and *β*_1_ parameters best fitting the data were -5.1 and 0.03 Gy^-1^, respectively. The probability of BRIA development was >10% per voxel for EQD2s >100 Gy (Fig 3). While most voxels had an EQD2<100 Gy, which would be typical for a high-grade glioma patient treated with 60Gy in 30 fractions, the logistic fit closely followed the empirical trend in probability of BRIA and dose-dependence up to an EQD2 of 150 Gy. Patient 2 was the only patient contributing to a cumulative voxel EQD2 >208.4. When Patient 2 was excluded from the analysis, the logistic fit parameters remained similar to those of the original model (*β*_0_ = −5.4 and *β*_1_ = 0.04 Gy^−1^). Fig 4 demonstrates our NTCP model’s estimate of the likelihood of BRIA development (green to pink scale) alongside the cumulative EQD2 map (blue to red scale), PTVs (outlined in white), and observed BRIA ROIs (outlined in black) for two representative patients in our cohort.

### Tumor progression and OS

Most (72%) patients had tumor progression. Of these, 10 (77%) progressed within BRIA ROIs. The median OS from initial BRIA observation was 8.9 months (range = 1.3–90 months) in the entire cohort (S2 Fig) and 8.4 months (range = 1.3–82.3 months) in patients with high-grade gliomas. One metastatic brain tumor patient died during the study period; this patient survived 2.1 months after BRIA observation. There was no difference in OS from BRIA appearance among patients with primary versus metastatic brain tumors (p = 0.2, log-rank test).

## Discussion/conclusions

The rising use of anti-VEGF therapy for brain tumors has led to BRIA detection in patients receiving radiation and bevacizumab. Histologic and imaging evidence have established these lesions as areas of coagulative necrosis with diffusion restriction and hypoperfusion on MRI, distinct from traditional radionecrosis [12, 22–24]. Nevertheless, patients with brain tumors receiving bevacizumab have often had tumor recurrence and multiple radiation courses [6], and frequently present with unclear imaging findings representing radionecrosis, BRIA, tumor progression, or a combination of the three. Appropriate patient management depends on accurate characterization of these entities, and improved understanding of the pathophysiology underlying BRIA occurrence may aid in distinguishing BRIA from these lesions. While our previous work demonstrated a possible link between radiation dose and BRIA development, the association between cumulative radiation dose and the likelihood of BRIA appearance has not been explored in a robust, NTCP analysis. We performed a voxel-based analysis of BRIA using diffusion and perfusion imaging along with cumulative voxel-specific radiation dose distributions. Our findings demonstrate that BRIA is most likely to occur in brain regions exposed to high cumulative radiation doses among patients with high-grade gliomas and brain metastases receiving radiotherapy and bevacizumab.

We identified a dose-dependent relationship between *cumulative voxel radiation dose* and BRIA appearance, with the probability of BRIA development >10% *per voxel* for EQD2s >100 Gy. In our previous work [12], we found that BRIA lesions occurred in brain areas receiving ≥60 Gy; yet, we did not perform an NTCP analysis of the association between cumulative radiation dose and BRIA development at the voxel level. Our model predicts that, on average, ≥10% of a volume receiving an EQD2>100 Gy will develop BRIA. This means that a 100 cc PTV receiving at least EQD2 of 100 Gy would develop an average BRIA volume of 10 cc or larger. EQD2 values >100 Gy are within the range of cumulative doses prescribed with reirradiation in glioblastoma, as well as treating with SRS (Fig 1). For example, 20 Gy in one fraction corresponds to an EQD2 of 110 Gy, assuming α/β = 2 Gy. Future studies correlating imaging and histopathologic findings in these patients will help to further characterize these lesions.

Previous studies have proposed a synergistic effect between radiation-induced vasculopathy and bevacizumab-mediated vascular pruning as a mechanism underlying BRIA development [11, 13]. In response to radiation-mediated vascular injury, tumor cells upregulate VEGF expression, precipitating the growth of leaky, tortuous vessels and compromising the BBB [25]. This promotes tumor hypoxia and contributes to radiation resistance. Bevacizumab acts as a radiosensitizer [26], normalizing the BBB and enhancing tumor oxygenation. Thus, BRIA may result from bevacizumab-mediated radiation hypersensitivity particularly apparent in brain regions exposed to the highest radiation doses. This is evident in Fig 4A, which demonstrates that the predicted probability of BRIA development was greatest in the area receiving the highest radiation dose. This area corresponded to the observed BRIA ROI (Fig 4B). Bevacizumab also reduces tumor blood vessel concentration and limits macrophages’ ability to clear necrotic cells filled with cytoplasm but lacking organelles [13, 27]. The restricted diffusion observed on DWI may therefore represent water diffusion limited by the relatively intact plasma membranes of these empty cells. This may explain why BRIA appears histopathologically as atypical coagulative necrosis, distinct from classic radionecrosis.

Despite these hypotheses, additional factors appear to contribute to BRIA development. Fig 4C highlights a patient who developed BRIA in one of three PTVs treated with similar doses simultaneously. Differential tissue-specific sensitivities to radiation within the brain have certainly been described [28, 29]. Alternatively, this phenomenon may result from distinct microenvironments within each PTV. Blood flow varies between brain regions due to edema, surgery and inflammation [30], meaning that bevacizumab exposure throughout the brain is likely nonuniform. Bevacizumab has a biphasic dose response; lower doses increase tumor blood flow, while intermediate doses produce the opposite effect [31]. The implications of this differential response for BRIA development are unclear. Tumor microenvironmental factors, including hypoxia, glucose deprivation, and lactic acidosis, confer radiotherapy resistance [32]. Reduced local anti-VEGF concentrations may increase blood flow and raise the risk of BRIA due to improved oxygenation and radiosensitivity. In other areas, higher local anti-VEGF levels may reduce blood flow, decreasing tumor oxygen delivery and the risk of BRIA. Ultimately, our results emphasize the complexity of BRIA’s pathogenesis. The interaction between bevacizumab exposure, tumor microenvironment, radiotherapy, and BRIA occurrence warrants further study.

While tumor oxygenation, glucose concentration, and pH are difficult to regulate, radiation dose may be manipulated to modify the likelihood of BRIA. This begs the question: what are the implications of BRIA for tumor progression and survival? Most patients in our cohort progressed in areas of BRIA. This suggests that BRIA may harbor active tumor cells. Previous studies have demonstrated that the size and stability of these lesions may correlate with survival [24, 33, 34]. The median OS from bevacizumab initiation among patients in this cohort mirrored that seen in other studies of patients with high-grade gliomas [35], with most patients dying in the first year after BRIA observation. Future studies assessing BRIA longitudinally are needed to clarify the significance of these lesions for patient outcomes and potential reirradiation, particularly among patients with brain metastases, who may undergo multiple courses of intracranial radiotherapy. These may influence radiation treatment guidelines in patients receiving bevacizumab.

This study has several limitations. This study was retrospective, and our sample size was small because we excluded patients without complete DICOM dose files for all radiotherapy courses. Nevertheless, we explored dosimetric predictors of BRIA development at the *voxel* rather than *patient* level. We performed a robust, voxel-based analysis with over 200 million voxels contributing dose, ADC, and DWI data, with validation of our registration accuracy. CT-MRI coregistration error was much smaller than the dose calculation grid size of 2.5mm, which supports our dose-response findings. We accounted for varying radiation courses and doses by calculating cumulative voxel EQD2 [17], which included combining conventionally hyperfractionated treatments. Future studies should explore patient-specific predictors for BRIA development in a larger population. We acknowledge that linear quadratic (LQ) formalism is inaccurate for large doses per fraction, as it overpredicts biological effect. Given that the true dose response as a function of radiation dose per fraction remains unknown, the LQ approach comprises a convenient method to combine radiation doses. We assessed BRIA at first appearance on imaging, rather than changes to BRIA over time. Further longitudinal investigation of BRIA characteristics may provide additional insight into BRIA’s pathogenesis and implications for survival. Duration and timing of bevacizumab exposure relative to radiation therapy should also be explored in future studies. Non-invasive multiparametric imaging is standard of care for establishing presence of BRIA in the clinical setting, thus we did not have pathologic confirmation of BRIA among patients in our cohort. However, BRIA was strictly characterized using both diffusion and perfusion MRI in accordance with methods previously defined by our group [13]. Nevertheless, we cannot completely exclude the possibility of mixed processes, including radiation changes and tumor progression, underlying these imaging findings. Larger studies with radiology-pathology correlations are needed to better characterize BRIA on imaging.

In this voxel-wise NTCP study, we show that BRIA development is radiation dose-dependent among patients treated with bevacizumab and radiotherapy. Our results may guide clinicians in evaluating areas of restricted diffusion in patients receiving these therapies. Additionally, our findings point towards microenvironmental factors as contributors to this process. Further studies that correlate radiology and pathology will aid in distinguishing BRIA from typical radionecrosis on imaging. Moreover, studies investigating the implications of BRIA for tumor progression and OS may guide radiation management and improve outcomes in this population.

## Supporting information

S1 Fig Timing of radiation courses and appearance of BRIA for each patient. Bar height represents time to BRIA appearance for each patient. Individual radiation courses are indicated by colored rectangles and are color-coded according to planning target volume. Prescription radiation dose for each radiation course is listed inside each radiation course box. Purple rectangles indicate initiation of bevacizumab, with purple outlines extending through duration of treatment. Patients with an asterisk (*) received at least one cycle of bevacizumab during a course of radiation.
(PDF)

S2 Fig Overall survival from first day of BRIA appearance for the study cohort. The grey shaded area represents the upper and lower bounds of the 95% confidence interval for the survival probability. Abbreviations: OS, overall survival.
(PDF)

## Abbreviations

ADC: apparent diffusion coefficient
BBB: blood brain barrier
BRIA: bevacizumab-related imaging abnormality
DWI: diffusion-weighted magnetic resonance imaging
EQD2: equivalent dose in 2 Gy fractions
NTCP: normal tissue complication probability
