# Supplementary figures and images for: The relationship between radiation dose and bevacizumab-related imaging abnormality in patients with brain tumors: A voxel-wise normal tissue complication probability (NTCP) analysis
Source: PLoS One. 2023 Feb 17;18(2):e0279812. doi: 10.1371/journal.pone.0279812 (PMC9937457; doi:10.1371/journal.pone.0279812)

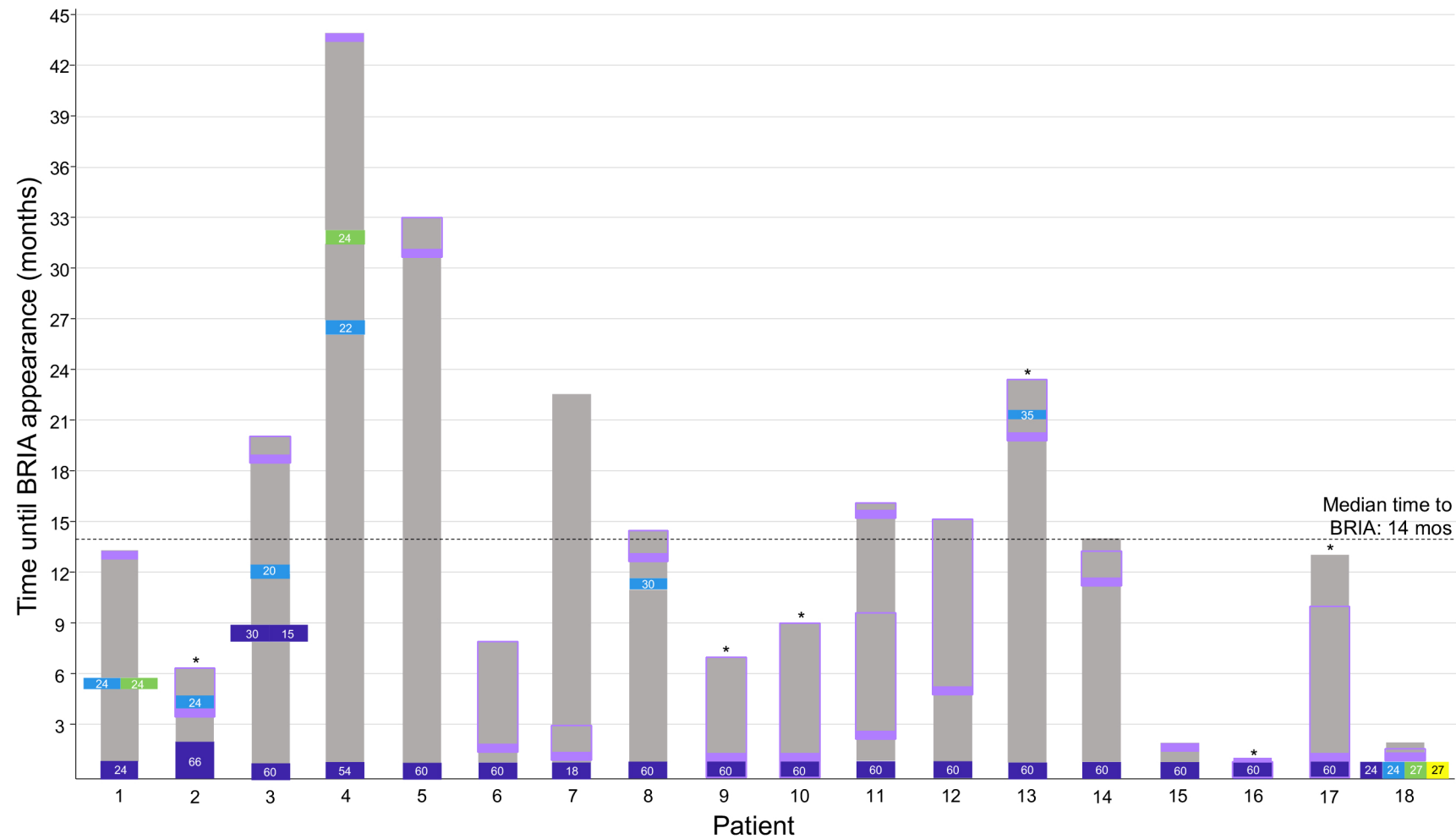

Supplement: S1 Fig — Bar height represents time to BRIA appearance for each patient. Individual radiation courses are indicated by colored rectangles and are color-coded according to planning target volume. Prescription radiation dose for each radiation course is listed inside each radiation course box. Purple rectangles indicate initiation of bevacizumab, with purple outlines extending through duration of treatment. Patients with an asterisk (*) received at least one cycle of bevacizumab during a course of radiation. (PDF) [file pone.0279812.s001.pdf]

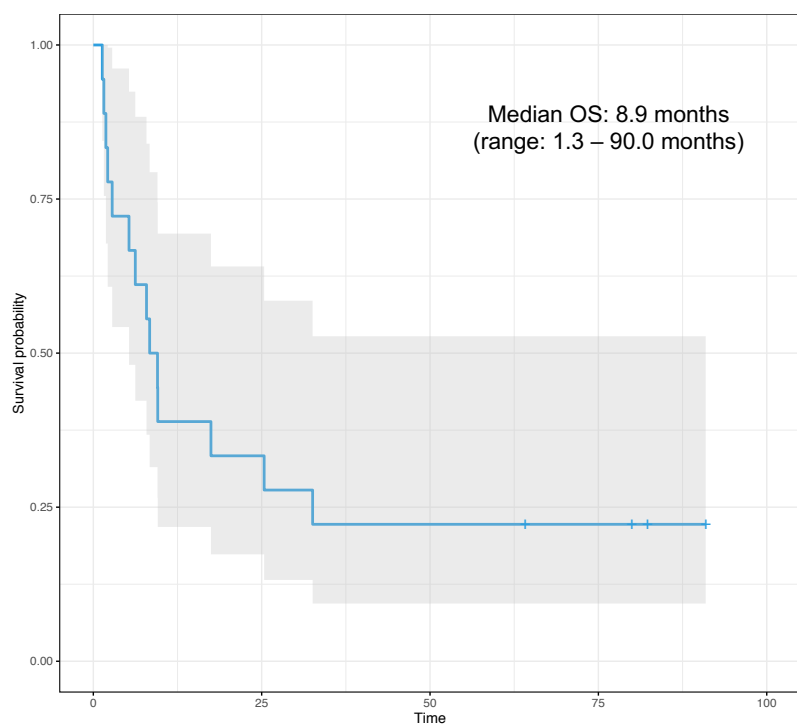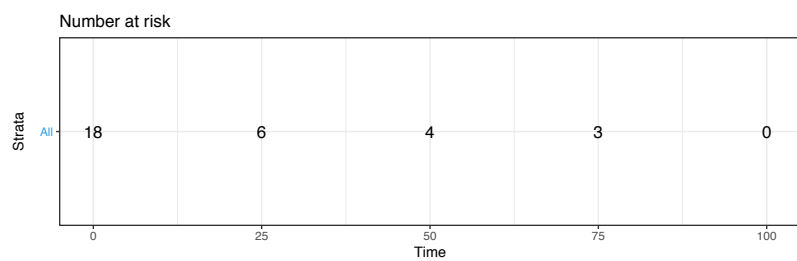

Supplement: S2 Fig — The grey shaded area represents the upper and lower bounds of the 95% confidence interval for the survival probability. Abbreviations: OS, overall survival. (PDF) [file pone.0279812.s002.pdf]
